# Sprayed Graphene-Based Coating Enabling Antifog and Smart Features

**DOI:** 10.1021/acsami.5c05680

**Published:** 2025-06-13

**Authors:** Antonio Alessio Leonardi, Teresa Berges Acosta, Hongyang Dang, Sihui Liu, Chengning Yao, Felice Torrisi

**Affiliations:** † Dipartimento di Fisica e Astronomia, 9298 Università di Catania, Via Santa Sofia 64, Catania 95123, Italy; ‡ CNR-IMM, Catania University Unit, Via Santa Sofia 64, Catania 95123, Italy; § Department of Chemistry, Molecular Sciences Research Hub, 4615 Imperial College London, London W12 0BZ, United Kingdom; ∥ Centre for Processable Electronics, Imperial College London, London SW7 2AZ, United Kingdom

**Keywords:** polyethylene, graphene, antifog, greenhouse, smart agriculture, agrifood, smart polymers

## Abstract

Large-area and low-cost coatings introducing electrical, optical, thermal, and surface control functionalities are highly demanded in the fields of flexible and wearable electronics for applications ranging from agrifood and smart packaging to electronic textiles and aerospace. Here, we report a graphene-based coating with combined antifog, electrical, and thermal functionalities suitable for spray deposition on commercially recyclable plastic substrates. A graphene ink formulation in a low-boiling-point solvent, stabilized by polyvinylpyrrolidone, enables compatibility with deposition processes in the coating industry. Spray-coated graphene films on polyethylene demonstrated antifog properties, achieving a 1.5-fold reduction in the contact angle at a 5% higher optical transmittance compared to commercial antifog coatings. Mechanical testing revealed more than a 1.8-fold improvement in Young’s modulus compared to the bare polyethylene. Moreover, by depositing another thin film of poly­(3,4-ethylenedioxythiophene)-poly­(styrenesulfonate) (PEDOT:PSS) underneath the graphene coating, electrical conductivity (sheet resistance of about 330 Ω/sq) with similar transparency can be achieved. Testing of the thermal properties of the film demonstrated how these conductive coatings can be used as heaters, reaching about 50 °C at a 30 V bias. Moreover, the graphene smart antifog coating showed a 97% improvement in thermal conductivity, increasing to 283% for the graphene/PEDOT:PSS multilayer coating with respect to the PE. The integration of these combined and novel smart functionalities on commercial recyclable plastic widens the application arena of smart coatings beyond agrifood, toward packaging, automotive, and aerospace fields.

## Graphical abstract

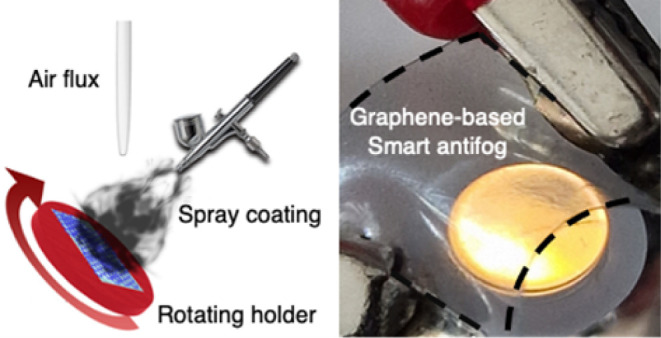

## Introduction

Advancements in coating technology have extended the inherent boundaries of materials, expanding the realms of design possibilities across various cutting-edge fields, and have unlocked new prospects. In recent years, smart coatings, characterized by their enhanced functionalities that go beyond traditional protection and exhibit active responses to specific stimuli, have emerged as highly versatile and bespoke solutions for various applications, including smart windows,^1^ deicing,^2^ corrosion protection,^3^ and more.

The agrifood sector with significant interest in antifog coatings for greenhouses represents an intriguing field of application for smart coatings. Numerous countries face challenges in ensuring year-round crop production due to extreme weather conditions in both cold and hot seasons, which are expected to worsen due to climate change.^4^ Greenhouses have assumed a significant role in modern agriculture by mitigating adverse weather conditions and enabling year-round crop cultivation.^5^

Successful greenhouse deployment hinges on various key design factors, and one of the most important requirements is the combines capability of surface thermal insulation and transparency in the visible range. However, the resulting temperature difference between the outside and inside of the greenhouse makes the formation of mist droplets (often referred as “fog”) on the greenhouse surfaces a serious issue. The abundant presence of water droplets can affect light transmission, influencing plant photosynthesis.^6^ Moreover, water droplets can act as lenses, burning plants and reducing crop yield.^7^

To avoid water condensation, a possible active strategy would be to strictly control the greenhouse parameters in terms of airflow, temperature, and humidity.^8^ However, maintaining a strict control of the environmental characteristics inside a greenhouse is complex and energy-demanding. On the contrary, a passive strategy would be to control the greenhouse materials by engineering the affinity between the surface and the water droplets, allowing for an antifog effect.^9^ The water droplet formation on the greenhouse surfaces and the droplets’ shape are influenced by the wetting properties of water on the greenhouse substrate and the substrate’s roughness.^10^ The contact angle (CA), defined as the angle where the interface between a liquid and vapor meets the solid surface, serves as the key determinant indicating the behavior of a liquid on a solid,^11^ making CA a crucial parameter for the design of materials or coatings for greenhouse panels. The higher the CA, the higher the radius of curvature of the drops on the greenhouse surface, meaning a higher angular dispersion of the light passing through the drops and, thus, overall higher optical losses.

Since the 1970s, polyethylene (PE) has become one of the most widely used materials for the construction of large-scale greenhouses for extensive agriculture due to its optical and mechanical properties^12,13^ and its relatively low cost (about 1 €/kg for low-density PE sheets during 2023).^14^ However, the hydrophobicity of PE characterized by a high CA of about 90°^15^ makes the polymer surface prone to the formation of water droplets, which generate fogging. This necessitates PE surface engineering to develop an efficient passive antifog strategy.^16^ The use of a hydrophilic coating to reduce the CA and light scattering is the most commonly employed strategy for antifogging.^17^

The first and most investigated route for commercial antifog is the use of migratory additives (tensioactives such as fatty ester acids, sorbitan, glycerol, polyglycerol, and so on) embedded into PE that, by migrating to the surface, reduce the surface tension, thus increasing the hydrophilicity.^18^ Concurrently, a small amount of the surfactant dissolves in the water droplets, resulting in a reduction in the surface tension of the water, causing the water to spread out into a thin layer across the film’s surface, thereby reducing sunlight scattering.^19^ This phenomenon is referred to as the “antifog” effect.^20^ The purpose of adding surfactants is not to prevent water from depositing onto the film but rather to condense it into an invisible, continuous, and thin layer.^18^

Some of the most commonly used additives are fatty acid esters, such as monoglycerides and polyglycerol esters,^21^ which are applied at a 0.2–3% weight concentration. The drawback associated with this strategy is ensuring a steady and long-lasting antifog effect, which, in the best case, is limited to a lifetime of about 2 years.^22^ Moreover, the use of additive migration species impacts the fabrication of multilayer films because the release profile in multilayer materials is also affected by the composition, arrangement, and thickness of the various layers.^23^ For these reasons, a second approach involving the use of coatings is receiving increasing attention as researchers constantly seek stable and robust alternatives.^16^ Roller processes and layer-by-layer fabrication are among the most investigated methods for antifog coatings.^24^ Several organic materials involving hydrophilic carboxyl/ester or hydroxyl groups such as polyethylene glycol, poly­(vinyl alcohol), acrylic acid, or natural water-soluble polymers were adopted recently to increase the hydrophilicity of polymeric films.^25,26^ In the case of a water-soluble polymers such as poly­(vinyl alcohol) (PVA), the interaction between their polar groups and water by hydrogen bonds favors the formation of a continuous water film with a low CA, thus reducing the scattering of light in the case of fog droplet formation. However, the natural water solubility of these polymers makes the films unstable over time and reduces the film’s mechanical properties. A recently emerging route toward antifog coatings has been the coupling of organic hydrophilic molecules, able to increase the antifog properties of greenhouse films, with inorganic materials mainly used to stabilize the organic counterpart in nano- or micro-network structures. Several examples of these strategies can be found in literature.^27,28^ Ref 27 tested different concentrations of PVA stabilized by SiO_2_ nanoparticles, improving the hydrophilicity of low-density PE (LDPE), achieving a CA that reaches super hydrophilic behavior (<8°) and that remains under 20° after 1 month, hence demonstrating good stability. Ref 9 applied a similar strategy by coupling sodium alginate and silica nanoparticles as a coating for PE, obtaining a CA of around 20° with stable CA and transmitted light when exposed to water mist for over 144 h at a temperature of 60 °C.

However, none of these studies demonstrated any additional functionalities enabled by these coatings. Multifunctional coatings that combine antifog properties with enhanced mechanical stability and electrical, and thermal conductivity open exciting opportunities for smart surfaces—not only for greenhouses but also across diverse fields such as electronics, construction, automotive, aeronautics, and aerospace. These findings are particularly significant given the mechanical stability of polymer substrates used outdoors and in harsh environments, where long-term durability is critical.

Graphene is a single layer of carbon atoms arranged in a hexagonal crystalline structure, and it is characterized by exceptional mechanical (Young’s modulus of up to ∼1 TPa), thermal (thermal conductivity of up to ∼5300 Wm^–1^K^–1^), electrical (mobility >250.000 cm^2^V^–1^s^–1^), and optical properties (optical transmittance, T, of up to ∼97.3% at 550 nm).^29–31^ Inks containing graphene and other 2D materials have already demonstrated high-performance printed electronics^32,33^ with devices manufactured on plastic,^34,35^ fibers,^36,37^ and textiles,^35,38–40^ enabling novel functionalities for flexible, wearable, and textile electronics,^41^ demonstrating the realization of several devices, such as a screen-printed capacitive touchpad on paper,^41^ a fully inkjet-printed integrated circuit,^35^ a strain sensor based on graphene fabricated directly on cotton fabric,^38^ and so on.

In particular, graphene is extremely interesting for the preparation of polymer composites characterized by enhanced mechanical strength combined with electrical conductivity and optical transparency, allowing truly smart packaging, and in general functional coatings that enable electrical and thermal conduction.^42,43^ Among all the graphene synthesis approaches, liquid-phase exfoliation (LPE) has been one of the most widely-used for the formulation of graphene inks. LPE is a well-established technique for the synthesis of large quantities of graphene and few-layer graphite (FLG) in a dispersion,^44^ stabilized by different surfactants or polymers such as polyvinylpyrrolidone (PVP)^34,45^ enabling the deposition of nanostructured thin films of graphene by spray coating^34^ and inkjet printing.^46^ Graphene flakes can be dispersed in an environmentally friendly, low-boiling-point solvent such as ethanol for a more sustainable formulation of graphene inks, avoiding toxic and harmful substances.^33,47^ Moreover, thanks to its vapor pressure, ethanol favors quick drying in inkjet printing and spray coating depositions. Graphene is known to be hydrophobic and so unsuitable to increase the hydrophilicity of a substrate.^48,49^ For this reason, graphene is only reported as an active defogger, involving the use of high-quality and large-area graphene sheets to enhance the evaporation of water mist by applying voltage (≤20 V).^50^ PVP is a widely used stabilizer in graphene inks but also a well-known hydrophilic polymer often used as an additive for hydrophilic membranes.^51^ We have already demonstrated the synthesis of high-quality graphene flakes stabilized with PVP.^52^

Thermal conductivity is a material property that measures how well a material conducts heat. The higher the thermal conductivity, the better the material is at transferring heat. Graphene with a hexagonal arrangement of carbon atoms on a single layer has the highest thermal conductivity (TC) among all known materials (>5000 Wm^–1^K^–1^, which is on par with diamond materials).^53^ Also, its 2D morphology and superhigh aspect ratio make it suitable as a filler for polymers to achieve exceptionally high TC.^54^ Researchers have demonstrated that graphene is a highly thermally conductive material and has been applied as a promising thermal filler to improve the thermal performance of the targeted material.^54^ Song et al.^55^ reported a thermally conductive polypropylene (PP)/graphene composite with an in-plane thermal conductivity of 10.93 Wm^–1^K^–1^, which is almost 55 times higher than that of pure PP. Yang et al.^56^ mixed reduced graphene oxide (rGO) into epoxy to improve its thermal conductivity. Their results show that the thermal conductivity of the composite coating reaches 1.239 Wm^–1^K^–1^, which is improved by 596% over pure epoxy.

In this work, we demonstrate a high-performance antifog coating based on graphene inks with additional electrical and thermal functionalities for advanced greenhouse applications. We propose a figure of merit of CA as a function of *T* as a metric to optimize the antifog properties of these coatings. Moreover, the stability to water exposure of our graphene-based antifog films was tested under a prolonged water spray jet. A graphene/poly­(3,4-ethylenedioxythiophene)-poly­(styrenesulfonate) (PEDOT:PSS) multilayer film is engineered and developed to introduce additional electrical and thermal functionalities to the antifog coating without losing the required transparency in the visible range. The use of graphene in combination with PEDOT:PSS is well-established in the literature, allowing the preparation of electrically conductive substrates (100–2000 Ω/sq) with optical transmittance in the visible range (76% transmittance at 660 nm)^34^ and thermoelectric properties.^57^ However, to the best of our knowledge, all the studies in literature report the deposition of PEDOT:PSS/graphene mixed inks on flexible substrates,^34,58^ while some multilayer structures have been demonstrated only for CVD-synthesized graphene.^59^ Hence, as far as we know, this is the first work reporting the use of a PEDOT:PSS graphene multilayer structure on a recyclable and flexible polymer using a graphene ink and a large-scale, and cost-effective approach such as the spray coating. The PEDOT:PSS graphene multilayer shown a remarkably low resistivity (about 330 Ω/sq, 5% lower *T* than the single graphene coating), opening the route towards smart functionalities such as electrical or improved thermal conductivity. Moreover, the electrical properties were demonstrated to fully recover after bending, with <10% resistance change for angles of <90°. The coating has shown a thermoelectric behavior that allows its use as a heater for temperatures up to about 50 °C with voltages up to 30 V. Moreover, thermal conductivity was also measured to demonstrate the improvement brought by graphene coatings, using the steady-state method (SSM) for the various samples. The single graphene antifog coating showed a 97% improvement in thermal conductivity, which increased to 283% for the graphene/PEDOT:PSS multilayer compared to the bare PE. The integration of novel smart functionalities widens the application arena beyond agrifood, toward packaging, automotive, and aerospace fields.

## Results and Discussions

### Fabrication and Characterization of the Graphene Coating

The graphene ink was prepared following previous reports^45^ and more details can be found in the Materials and Methods section. Graphite flakes were dispersed in an ethanol solution with a concentration of 10 mg/mL, along with 1.2 mg/mL of polyvinylpyrrolidone (PVP) as a stabilizing agent. The dispersion was subsequently introduced into a glass tube and subjected to sonication in an ultrasonic bath at a temperature of 10 °C for a duration of 9 h. Afterward, the solution underwent centrifugation for 1 h at 1400 RCF (Materials and Methods). The upper 80% of the dispersion was collected, and the graphene concentration was determined to be approximately 0.25 mg/mL by measuring the absorption at 660 nm via a UV–vis spectrophotometer and applying the Beer–Lambert law^44^ (Materials and Methods). This resulting ink was utilized for manufacturing the graphene-coated PE (G-PE) samples by spray coating. The graphene ink was subjected to a 10 min sonication prior deposition.

Ethanol was selected because it is an ideal solvent for spraying as it has a high evaporation rate, a low boiling point, and it is classified as a nontoxic solvent.^33,34,47^ PVP is a well-established stabilizing agent for graphene inks, avoiding flake agglomeration and precipitation.^34,45^ In this study, we prepared a graphene ink including PVP as a stabilizer (named simply as graphene inks in the manuscript) as an antifog coating able to lower the CA of PE without affecting the transmittance in the visible range. Spray coating was selected as the deposition approach thanks to its suitability for cost-effective thin-film deposition, and large-scale production.^60^

The spray coating of this ink and other similar graphene-based inks has already been conducted, demonstrating the feasibility of this deposition approach.^34,52^ The used graphene ink has already been characterized^45^ showing an average flake thickness of 5 layers, suggesting that the graphene ink consists of a mixture of single-layer graphene (SLG) and a limited number of layers in the graphene flakes (FLG).

Before spray coating, the sample was treated by a 10 min UV-ozone process to increase the wettability of the PE surface and the adhesion of the graphene coating. Spray coating was performed using the setup illustrated in Figure 1a and described in detail in the Materials and Methods section. Different samples of graphene-coated PE (graphene antifog-treated PE) were obtained by spraying graphene ink for different times: 5 s, 15 s, 30 s, 45 s, 1 min, 1 min 30 s, 2 min, 3 min, and 5 min.^61^ After spray coating, the samples were photonic annealed by a pulse light source (spectral range 530–1400 nm) with <1.5 ms flash duration and a dose of 2.5–5 J/cm^–2^ for 3 repetitions to increase the stability of the coating.^52^ This process can be found in some of our previous work to increase the conductivity of the samples without lowering their quality^45^ and it is used in the case of thermally sensitive substrates such as PE (melting temperature between 90 and 110 °C). Indeed, the idea of the process is to have a local thermal annealing limited to the surface without affecting the substrate, carbonizing the PVP, and probably increasing the graphene adhesion as an effect of the surface polymer softening. The graphene-coated PE films that underwent the photonic annealing are named G-PE for simplicity throughout the manuscript. A representative 30 s G-PE is shown in Figure 1b.

**Figure 1:**
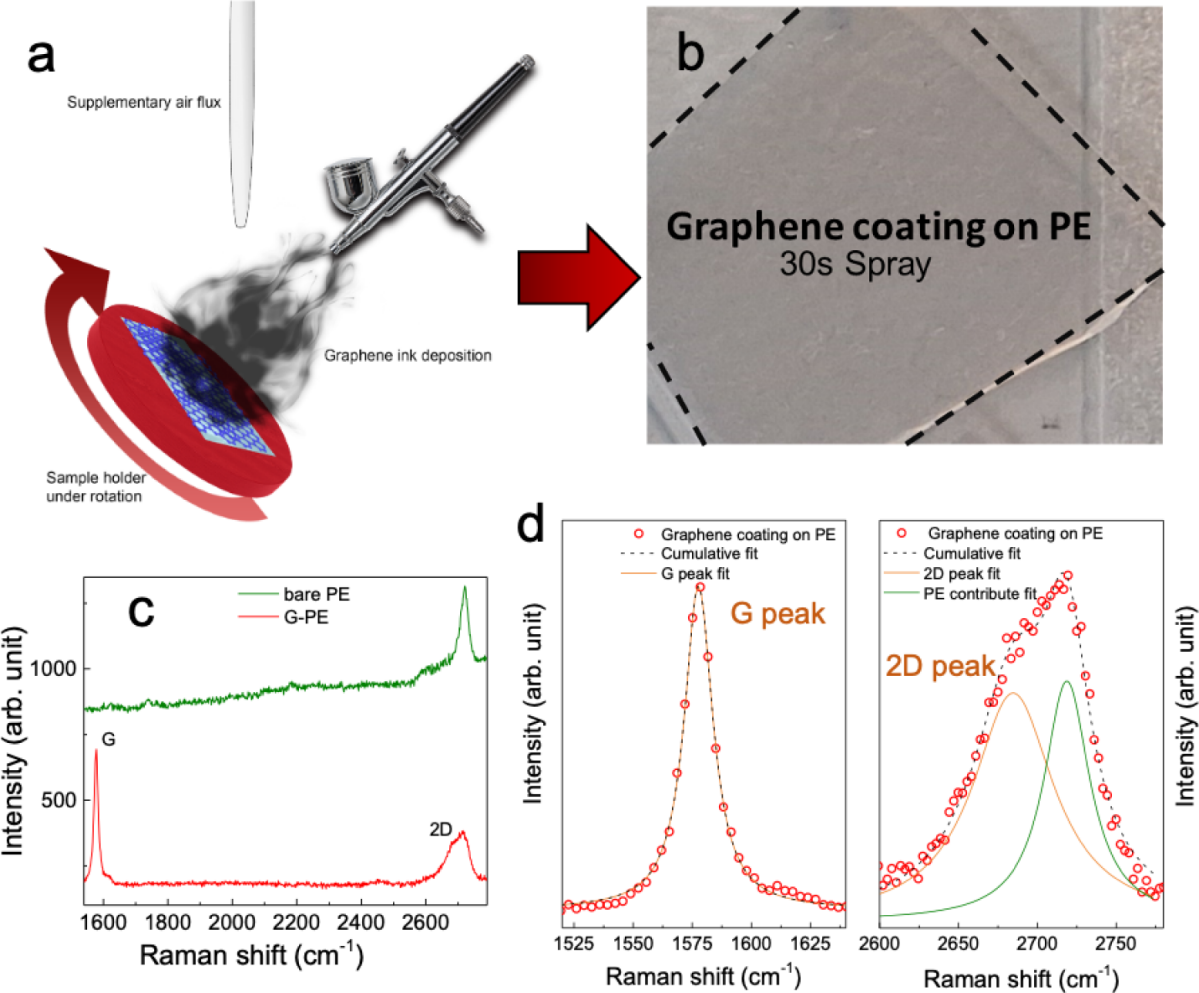
(a) Schematic representation of the graphene ink spray coating system. (b) Photo of a graphene-coated PE substrate. (c) Raman spectra of bare PE (green) and graphene–PVP-coated PE sample (red). (d) Raman fit of the G and 2D peaks (orange) deconvolving the PE contribute (blue).

Figure 1c shows the Raman spectra of both the pristine PE substrate (green) and the G-PE (in red) sample. We selected the 1500–2800 cm^–1^ spectral range due to the Raman shift associated with the graphene G and 2D Raman peaks.^35^ The spectra of G-PE show the typical G and 2D peaks^62^ at 1580 cm^–1^ and 2680 cm^–1^ respectively, demonstrating the effective deposition of a graphitic film. Figure 1d shows the Lorentz fit of the G and 2D Raman peaks shown in Figure 1c, where the 2D peak best fit is obtained by including the presence of the PE peak at 2720 cm^–1^in the peak deconvolution. The ratio between the G peak and the 2D one is around 0.92, confirming the presence of both SLGs and FLGs inside the ink, as previously reported.^45^

The Raman measurements align with the values previously reported for LPE graphene inks,^35,46^ demonstrating both the SLG and FLG high quality and confirming that the photonic annealing does not cause any damage to the flakes.^52^

### Antifog Properties

As discussed in the Introduction section, optical transmittance in the visible range and CA are crucial parameters of antifog-coated substrates for greenhouse applications. We aim to obtain graphene-PVP coatings presenting a higher or equal transmittance in the visible range, and a lower CA than that obtained by commercial antifog treatment on PE. Visible transmittance and CA were acquired for all of our coatings as a preliminary step to obtain the corresponding figure of merit (FoM), where we used the optical transmittance in the visible range and the CA of a commercial antifog-treated PE as a benchmark.

First of all, optical transmittance measurements were carried out for all the G-PE samples to find a range of optimum deposition time resulting in lower visible optical losses than those of commercial antifog-coated PE. UV–vis transmittance (*T*) measurements were carried out in the UV–vis-NIR range from 200–800 nm on the G-PE samples as a function of deposition time, ranging from 5 s to 5 min in Figure 2a. It is worth noting that the photonic annealing produced negligible transmittance variation compared with the untreated samples. In the same figure, it is possible to appreciate the *T* measurements of the bare PE substrate (in black) used as references, with about 74–84% *T* in the visible range (84% at 660 nm). The UV-ozone treatment applied before the spray coating produced a negligible *T* variation compared to the bare untreated PE substrate and so it is not reported. As it is possible to observe, for deposition time shorter than 30 s, only a ≤4% *T* decrease is observed compared to the uncoated PE. The inset of Figure 2a shows the transmittance variation as a function of deposition time in the visible range (400–700 nm). The absorption contribution of the graphene coating is featureless, and the transmittance decreases as the deposition time increases, following the same trend of uncoated PE as a function of the wavelength. Considering the interest of the transmitted light in the visible range for greenhouse application, we measured the integrated transmittance in the range of interest 400–700 nm to quantitatively attest the transmittance variation as a function of the deposition time. In Figure 2b, this integrated transmittance normalized to the one obtained for the bare PE film is reported as a function of the spray coating time (black dots). After 5 s of spray coating, only a 1% *T* decrease is observed, while after 15 s, the decrease reached 2%. After a 30 s deposition, *T* decreases by 4%, while for 45 s, *T* decreases by 5%. After 1 min of spray coating,*T* continues to decrease by approximately 5% every additional 30 s up to 2 min. Finally, for a 3 and 5 min deposition, a 22% and a 34% decrease in *T* is obtained, respectively.

**Figure 2:**
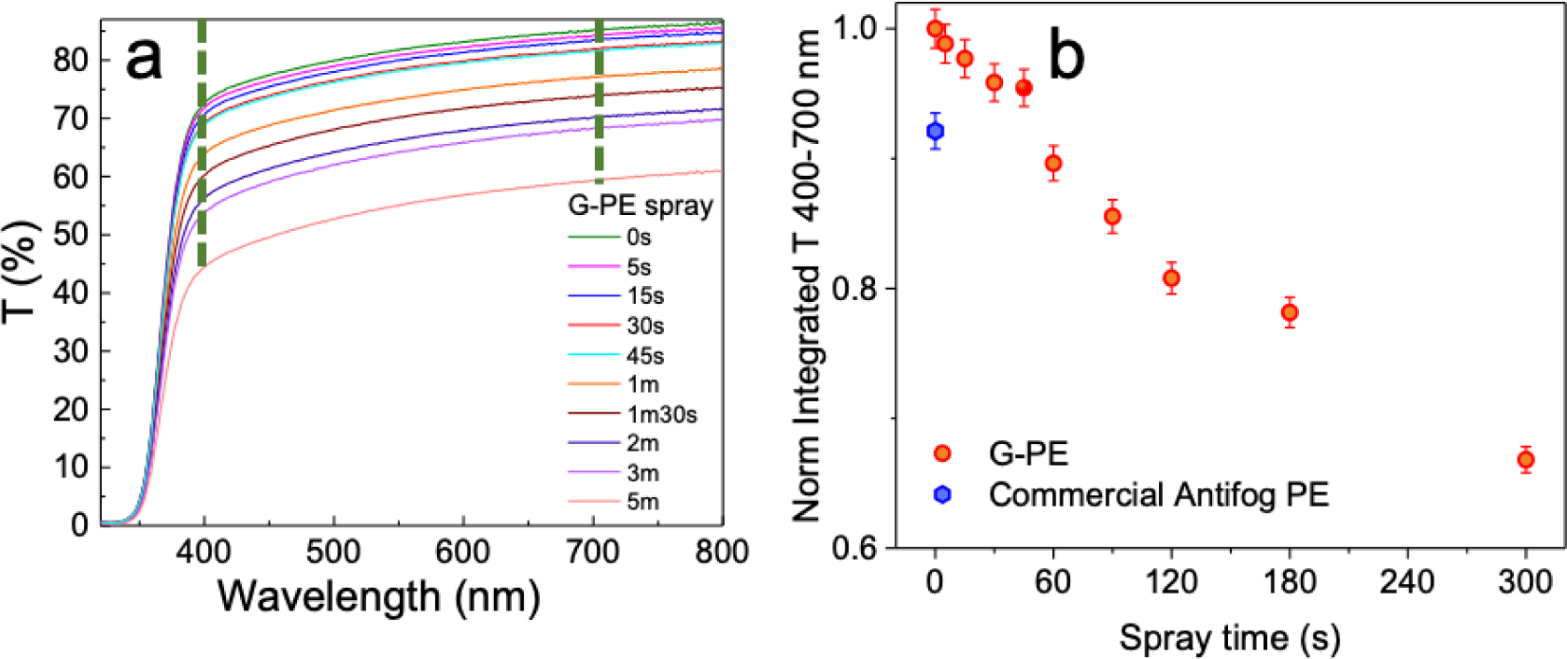
(a) UV–vis transmittance spectra obtained as a function of the spray coating deposition time. In the inset to (a) the 400–700 nm range is reported. (b) Integrated transmittance in the 400–700 nm range normalized to bare untreated sample (*n* = 3 points for each sample) as a function of the spray coating time.

The normalized integrated transmittance value measured for the commercial antifog PE film (blue curve) is used as a benchmark to identify a range of deposition times for the graphene-PVP coating with higher transmittance in the visible range. The commercial antifog sample showed an 8% decrease in *T* compared to the bare PE film. As it is possible to observe, the G-PE films with spray coating times up to 45 s resulted in a higher normalized integrated transmittance than the commercial antifog PE film. On the other hand, spray-coating times longer than 60 s resulted in normalized integrated *T* values, lower than those of commercial antifog coating.

Static CA measurements were carried out to study the wettability of the G-PE films as a function of the deposition time. Figure 3a shows the images taken for the CA measurements of bare PE, commercial antifog PE, and 30 s G-PE are shown. The CA changes from 90° for the uncoated PE to ∼70° for the commercial antifog sample, and to about 45° for a 30 s G-PE film. All of the static CA measurements performed on graphene-coated PE films, with and without photonic annealing as a function of deposition time, are reported in Figure 3b. The bare PE sample showed a 90° CA (in green), while a CA of about 70° was obtained by the commercial antifog PE film (in blue). The CA progressively decreases in the graphene-coated PE samples, going from CA ∼60 ° ± 4° (for both untreated samples in black and photonic annealed samples in red) for 5 s of deposition to 42° ± 3° and 48° ± 3° for 15 s of deposition in the untreated and pulsed light-treated samples, respectively. Increasing the spray coating time to 30 s yielded a CA of 44° ± 3° and 45° ± 3° for the untreated and pulsed light-treated samples, respectively. Beyond 30 s deposition of the graphene ink, the CA seems to plateau at ∼40–50°. As observed, in most cases, the CA does not change significantly between the photonic annealed samples and the untreated ones, with a slightly higher CA for the treated samples.

**Figure 3:**
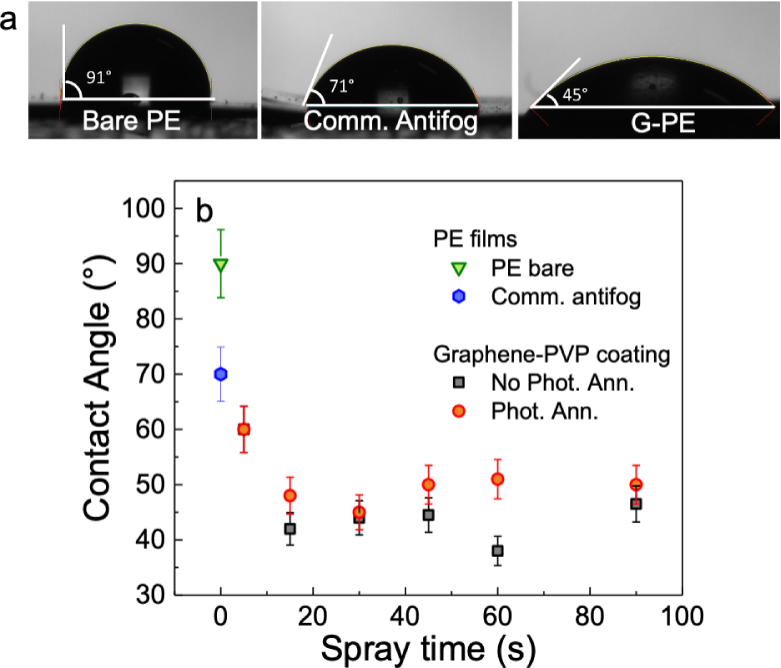
(a) Contact angle measurement on a bare PE, commercial antifog PE sample, and 30 s graphene-coated sample (with photonic annealing). (b) CA as a function of the spray coating time for the tailored graphene ink with and without the photonic annealing. In the same graph, the CA obtained from PE bare and commercial antifog PE are reported in green and blue, respectively. All measurements refer to *n* = 3 points for each sample.

A detailed investigation of the mechanisms underlying changes in the hydrophilic properties of the PE substrates as a function of graphene and PVP (used as stabilizers) concentrations was beyond the scope of this work. As noted in the Introduction section, several studies have reported the use of organic hydrophilic molecules to enhance the antifog properties of greenhouse films, with inorganic materials serving to stabilize the organic component. We adopted a similar strategy, using the graphene coating as a stabilizer for PE, which we believe is primarily responsible for the hydrophilic character of the coating, given graphene’s intrinsic hydrophobicity.

The characterization of transparent antifog coatings requires an appropriate FoM that evaluates both wettability and optical properties. We identify as a suitable FoM assessing the quality of transparent antifog coatings: the CA as a function of the integrated transmittance in the 400–700 nm optical range, normalised to that of the untreated PE substrate. The resulting FoM is shown in Figure 4a where G-PE photonic-annealed films are reported in red for different deposition times, while the uncoated PE and the commercial antifog PE film are reported as a benchmarks in green and blue, respectively. We can identify four different quadrants in the graph (as defined by the horizontal and vertical blue lines) based on a comparison between the G-PE data and the commercial antifog PE film. The top-left quadrant includes samples with higher *T* but lower CA (uncoated PE film), while the top-right quadrant includes samples with higher CA and higher *T*. In these two quadrants, no G-PE samples are present. On the bottom-right, we have samples with lower *T* but higher CA (G-PE with spray deposition >30 s), while on the bottom-left, we include samples with lower CA and higher *T* (G-PE with spray deposition <30 s). G-PE sample with spray deposition time between 5 s and 30 s fall in the quadrant of higher *T* and lower CA than the commercial antifog PE film, demonstrating potential as a transparent antifog coating for PE. We then select the G-PE sample with 30 s graphene ink deposition time, as it maximizes both CA and T while minimizing the spray coating time.

**Figure 4:**
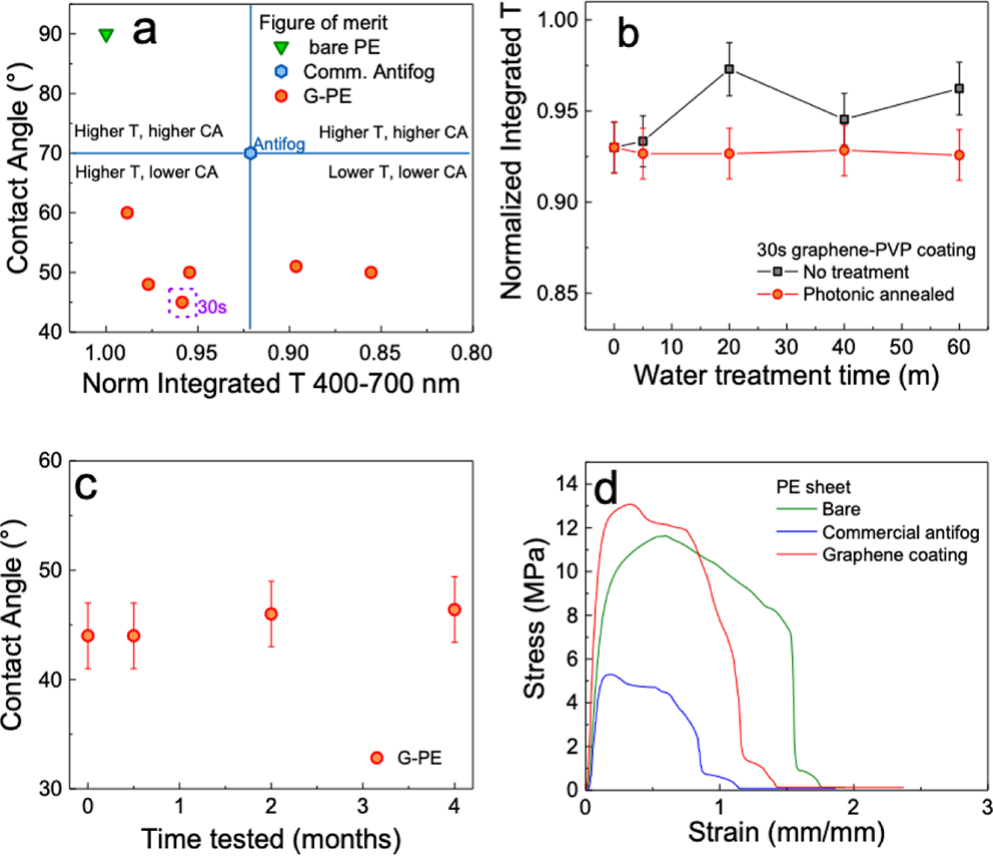
(a) Contact angle as a function of integrated transmittance in the 400–700 nm range normalized to the bare PE of the G-PE samples reported as a figure of merit. (b) Water resistance test in terms of variation of the integrated transmittance in the 400–700 nm range normalized to the bare PE as a function of the water exposure (*n* = 3 points for each sample). (c) Contact angle measurement as a function of time (*n* = 3 points for each sample). (d) Tensile tester measurement of the bare PE sheet (red), standard commercial antifog (blue), and our 30 s graphene spray coating treatment (black).

Stability of the antifog coating when in contact with water is a crucial point for this application. Normally, graphene and PVP can be easily dispersed in water.^63^ Photonic annealing has already been proven as a successful method for improving the stability of a graphene coating in an aqueous solution.^52^ However, to further prove the G-PE stability in contact with water, we designed a specific test consisting of spraying water for up to 1 h, while regularly checking for any transmittance variation. In this test, the objective was to measure the transmittance of the samples to determine whether any changes had occurred. In the case of an unstable coating, the graphene flakes would redisperse in water. Therefore, if the transmittance change is negligible, it indicates that there was no redispersion and that the coating remains stable when exposed to water.

Samples coated with graphene, with and without photonic annealing, were tested (more details in the Materials and Methods section). Figure 4b shows the integrated transmittance (in the spectral range of 400 nm to 700 nm), of the graphene G-PE normalised to that of the bare PE substrate In particular, the *T* as a function of the water spray time is reported (Figure 4b) in red for the G-PE with the photonic annealing and in black for the same coating but without the photonic annealing. For the graphene coating without the photonic annealing, an increase in the integrated transmittance is attested starting after 20 min of water spray exposure, while for the photonic-annealed samples, the transmittance variation is negligible even after 1 h.

The stability over time of the wettability induced by the graphene coating was also investigated. In particular, the CA was monitored as a function of time (from as-prepared to 4 months after) for the G-PE, as reported in Figure 4c. The CA of 44° ± 3° for the as-prepared G-PE sample remained unchanged after 2 weeks and slightly changed to 46° ± 3° after 2 months, reaching 47° ± 3° after 4 months. However, this slight variation is well within the error bars. Long-term resistance and performance retention are critical factors for antifog applications, especially in outdoor or harsh environments. In this study, we simulated water resistance (Figure 4b) and aging (Figure 4c) to demonstrate that the proposed coating is a promising candidate for prototypal testing. While long-term performance validation in real-world applications and assessments beyond technology readiness level (TRL) 5 are outside the scope of this work, the significant antifog properties, smart functionalities, aging, and durability under water exposure of our graphene coating make it a compelling subject for future research in relevant environments. The long-term resistance of these coatings induced by proper optimization of the graphene deposition may open routes to several outdoor applications besides antifog for greenhouses, including building construction, automotive, and aerospace. Adhesion tests were also performed following the ISO 2409 standard, resulting in an excellent adhesion classification of 1, demonstrating the coating’s robustness (more information in the Supporting Information).

We also investigated the mechanical properties of the G-PE, as shown in Figure 4d. In particular, this figure shows the measurement of stress as a function of strain performed by a tensile tester (Materials and Methods) for a bare PE (red curve), commercial antifog PE film (blue curve), and a 30 s G-PE (black curve).

The resulting Young’s modulus, yield strength, and ultimate tensile strength for these samples were obtained from the stress vs strain curves and are reported in Table 1. All the measured mechanical properties worsened in the commercial antifog film compared to the bare PE. For the commercial antifog PE film, the Young’s modulus, yield strength, and ultimate strength decreased by 1.6, 2, and 2.3 times, respectively, when compared to the bare PE. On the other hand, a significant improvement is attested in the case of the graphene coating with respect to the bare PE, with a 1.8-, 1.4-, and 1.1-fold increase. This improvement is even more significant compared to the commercial reference, with an increase in the Young’s modulus, yield strength, and ultimate strength of 3, 2.8, and 2.6 times, respectively. An increase in Young’s modulus indicates that the material becomes more rigid and less flexible, enhancing its resistance to stretching. As a result, the substrate can endure longer periods of use without experiencing damage or breakage. Yield strength is the stress level at which a material begins to permanently deform. Beyond this threshold, the material fails to return to its original shape once the stress is removed, signifying the shift from an elastic to plastic deformation. An improvement in yield strength means that the sample can withstand higher stress without permanent deformation, thus preserving its elastic behavior for a longer period. Furthermore, an increase in ultimate tensile strength implies that the material can endure greater stretching forces before breaking and becoming unusable. The overall improvement in the mechanical performance is of great interest, considering the outdoor use of antifog films, where durability is essential for prolonged use.

**Table 1:** Tensile Tester Results

Sample	Young’s modulus [MPa]	Yield strength [MPa]	Ultimate strength [Mpa]
PE bare	59 ± 2	8 ± 0.2	11.5 ± 0.2
Commercial antifog PE film	36 ± 2	4 ± 0.1	5 ± 0.1
30 s G-PE	108 ± 5	11 ± 0.3	13 ± 0.3

### Smart Antifog Coating of Graphene/PEDOT:PSS Multilayer

We prepared a transparent smart antifog coating, using a graphene/PEDOT:PSS multilayer structure. The graphene/PEDOT:PSS-coated PE (G/P-PE) was created by spray-coating PEDOT:PSS ink for 1 min and 30 s on PE substrates (more details in Materials and Methods), followed by 30 s spray coating of graphene ink (given this showed the best results in terms of FoM).

The G/P-PE is prepared as described in the Materials and Methods section. The PEDOT:PSS layer resulted in a sheet resistance of about 600 Ω/sq while the graphene/PEDOT:PSS multilayer showed a sheet resistance of 330Ω/sq (see Materials and Methods). The PEDOT:PSS conductive layer reduced *T* of the graphene by just 5%, while increasing the conductivity of the G/P-PE film. The sheet resistance of the PEDOT:PSS-coated PE and G/P-PE was measured by the Van der Pauw approach, and more details on the used equipment is available in Materials and Methods section. The comparison of the sheet resistance of G/P-PE with values in literature is reported in Table 2, showing that the obtained value is one of the best for solution processed inks, considering that a similar value was obtained in ref 59 using CVD-grown graphene.

**Table 2:** Sheet Resistance

Material	Type of structure	Fab. method	Sheet resistance [Ω/sq]	Ref.
PEDOT:PSS	Single layer	Spray coating	570 ± 30	This work
G/P-PE	Multilayer	Spray coating	330 ± 40	This work
Graphene/PEDOT:PSS	Single layer	Spray coating	2000 ± 500	^34^
Graphene/PEDOT:PSS	Single layer	Spray coating	500	^58^
Graphene/PEDOT:PSS	Multilayer	Graphene CVD, PEDOT:PSS Spray coating	300	^64^
Graphene	Single layer	Screen printing	2400	^59^

In particular, the obtained sheet resistance of 330 Ω/sq is lower than the 2000 Ω/sq previously obtained by our group and the 500 Ω/sq by ref 58, both using a graphene/PEDOT:PSS mixture and considering a similar transmittance (about 76% at 660 nm).

Electrical and mechanical characterizations of the film are shown in Figure 5. Figure 5a shows the I–V curve of the G/P-PE (structure schematic in the inset). The curve is clearly linear, confirming the ohmic behavior of the coating. Mechanical bend test results are shown in Figure 5b. The samples were tested after repeated bending between −180° and 180° angles for than ten times. The bending test performed on these samples showed less than 15% change in normalized resistance, for angles lower than 90° and most importantly the full recovery of the initial resistance. The inset of Figure 5b shows a photo of an LED connected by the G/P-PE. The sample retains transparency and flexibility upon bending, while providing power to the LED.

**Figure 5:**
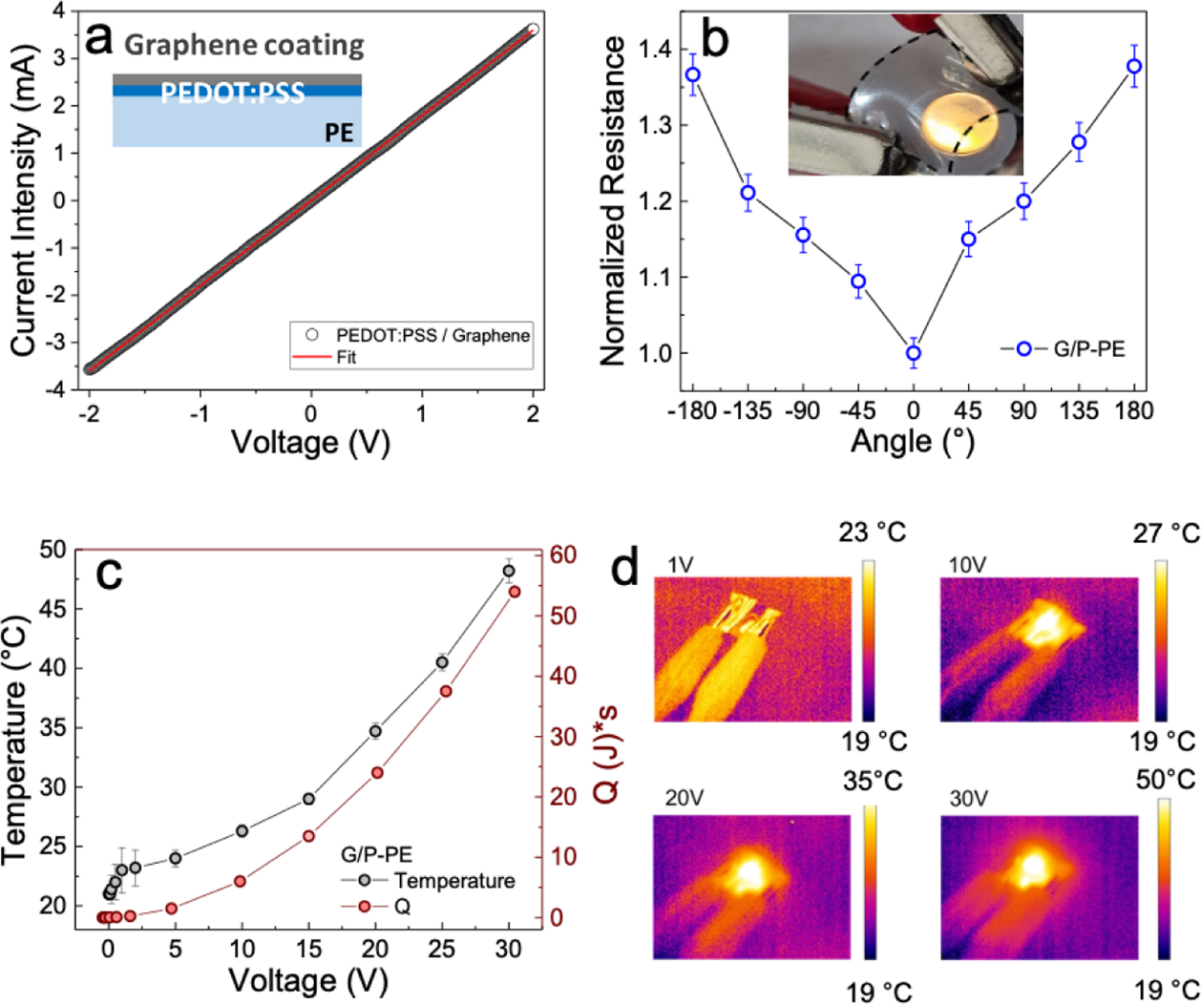
(a) Current intensity as a function of the voltage (I–V curve) for the multilayer graphene/PEDOT:PSS coating. In the inset to (a), a scheme of the multilayer structure is reported. (b) Normalized resistance measurement as a function of bending (measured over *n* = 10 bending cycles). In the inset to (b), a photo of an LED connected by the conductive, transparent, and flexible G/P-PE film. (c) Temperature (in black) and dissipated heat (in red) are reported as a function of voltage. (d) Thermocamera images acquired during the thermoelectric test at different voltages.

We finally tested the the G/P-PE samples as heaters, investigating their thermal behaviour. Low-temperature (<100 °C) heaters can be used for deicing or active defogging applications. For this reason, we characterized the heating properties of these G/P-PE by biasing them with a power source at different voltages, while simultanously acquiring their IR images and temperatures with a FLIR thermocamera. In Figure 5c, both the temperature (black) and dissipated heat (red) as a function of the voltage are reported. In particular, the dissipated heat was obtained according to eq 1, with a dissipation time of 3 min for each point.

1 $$Q=\frac{V^{2}}{R}\times t$$

In Figure 5d, the acquired thermocamera images are reported for different voltages. This shows that, apart from electrical conduction, these plastic substrates can be used as heaters in packaging or agrifood applications.

The temperature trend as a function of the voltage is very similar to what is observed in the literature for similar cases.^65^ However, as shown in Figure 5c, at voltages lower than about 2 V, a non linear trend for the current is observed, corresponding to a nonuniform temperature observed by the thermocamera, as also visible in the 1 V image in Figure 5d. We attribute this behaviour to a uniformly distributed voltage along the film, as the temperature map seems suggesting, and this causes the presence of a few hot spots with a rapid increase in the temperature as a function of the voltage. At higher voltages, this sort of percolation regime breaksdown, resulting in a more gradual temperature increase. At 5 V, a temperature of 24 °C is achieved, increasing to 26.3 °C at 10 V and 29 °C at 15 V. At 20 V, the temperature reaches 34.7 °C, going to 40.5 °C at 25 V. Finally, a maximum temperature of 48.2 °C is obtained at 30 V. To extractan accurate Seebeck’s coefficient, a more extended range should be considered. However, within the tested range, the average Seebeck coefficient was about 596.5 μV/K.

### Thermal Conductivities of G-PE and G/P-PE

The SSM was performed with a physical property measurement system (PPMS, Quantum Design) using a thermal transport option (TTO). In the SSM method, the sample was heated with a known steady-state heat flux (*q*), and the resulting temperature drop (Δ*T*) across a given length (Δ*x*) of the sample was recorded after the system reached thermal equilibrium, allowing the calculation of the thermal conductivity (*κ*) as *κ* = −*q*Δ*x*/Δ*T*. Emissivity values of 0.93, 0.80, 0.88, and 0.85 were applied for the PE, graphene/PE, PEDOT:PSS/PE and for graphene/PEDOT:PSS/PE samples, respectively, to estimate radiative losses at the room temperature. Key geometry parameters are shown in the Supporting Information.

At room temperature (∼303 K), bare PE exhibits a *κ* of 0.419 ± 0.150 Wm^–1^K^–1^, consistent with reported values^66–68^ (Figure 6a). The PEDOT:PSS and graphene coatings on the PE sheets improve the *κ* by 63.2% to 0.684 Wm^–1^K^–1^ and by 97.1% to 0.828 Wm^–1^K^–1^, respectively. The combined layer coating of graphene/PEDOT:PSS on the PE sheets shows the highest *κ* values at 303 K (1.606 Wm^–1^K^–1^), revealing an enhancement in the thermal conductivity of up to 283.3%, compared with that of the bare PE sheets. When comparing the individual contribution of each coating, the PEDOT:PSS and the graphene layers improve *κ* by 94.0%, and 134.8%, respectively, indicating that the graphene layer plays a more important role in providing thermal conductivity.

**Figure 6:**
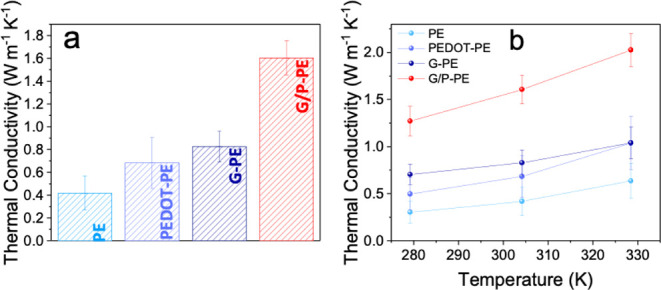
(a) Comparison of the κ values between the PE, G-PE, PEDOT:PSS/PE, and G/P-PE samples at around room temperature using the SSM method (*n* = 3 points for each sample). (b) Temperature-dependent thermal conductivity (κ) values of bare PE, PEDOT-PE, G-PE, and G/P-PE films, measured at 278, 303, and 328 K using the SSM method (*n* = 3 points for each sample). The G/P-PE film exhibits the highest κ, and the steepest increase with temperature, surpassing 2 ± 0.4 Wm^–1^K^–1^ at 328 K.

To investigate temperature-dependent behavior, *κ* was also measured at 278, 303, and 328 K (Figure 6b). Across all samples, *κ* increases with temperature, with the G/P-PE film exceeding 2 Wm^–1^K^–1^ at 328 K. This sample also exhibits the steepest temperature dependence, emphasizing the complementary effects of PEDOT:PSS, which enhances interfacial phonon transport, and graphene, which provides efficient conduction networks, particularly at elevated temperatures.

These findings demonstrate the potential of these antifog smart coatings to significantly enhance thermal management in polymer-based composites, offering promising applications in flexible electronics and energy systems.

## Conclusion

We demostrate an improved antifog graphene coating and a smart multilayer PEDOT:PSS/graphene coating on polymer substrates. All of the coatings were obtained by an optimized spray coating system that represents a cost-effective and industrially compatible method. The graphene coating showed a reduction in contact angle by about 2.25 times compared to the original 90° of PE. A novel figure of merit has been introduced for this application, reporting the CA as a function of the integrated transmittance in the 400–700 nm range, normalized to the pristine untreated material, showing the optimization of both CA and visible transmittance. The graphene coating showed a 5% higher transmittance compared to a typical commercial antifog treatment of the same PE. Tensile testing demonstrates a 1.8-fold greater Young’s modulus compared to the bare PE and a strong coating adhesion under multiple bending cycles. The stability of the resulting antifog films was tested under heavy water exposure, demonstrating a negligible variation of their properties after the test. A sheet resistance of 330 Ω/sq was obtained by the sample with ideal recovery after bending. Notably, the smart coatings lead to an enhancement of the thermal conductivity, with 97% and 283% improvement for graphene and graphene/PEDOT:PSS multilayer coatings, respectively. These results may lead to electrical and thermal functionalities in recyclable polymers for different application fields, such as agriculture, packaging, defense, and aerospace.

## Materials and Methods

### Materials

Bare PE sheets and PE sheets with antifog treatment were obtained from Bandera. Graphite flakes (product number 332461), ethanol (ACS reagent grade), and polyvinylpyrrolidone (average Mw = 40,000) were acquired from Merck. PEDOT:PSS aqueous solution was purchased from Heraeus (Clevios PH 1000). Deionized water (18 MΩcm) was obtained using a Millipore Milli-Q system.

### Graphene Ink Preparation

A 40 mL ethanol dispersion of 0.4 g of graphite with 1.2 mg/mL of PVP as a stabilizer was initially prepared. The dispersion was then loaded into a glass tube and sonicated in an ultrasonic bath (Fisherbrand Elmasonic S150) at 10 °C for 9 h. The temperature was set as constant thanks to the use of a chiller. The solution was subsequently centrifuged for 1 h at 1400 RCF (Allegra 64R centrifuge). The top 80% of the dispersion was collected, and its concentration was found to be around 0.25 mg/mL by measuring the absorption at 660 nm through a UV–vis measurement and using the Beer–Lambert law.^44^ The viscosity and surface tension of our inks are 2.35 ± 0.1 mPa·s and 18.50 ± 0.25 mN/m, respectively.^52^ The obtained ink was used to fabricate the graphene coating by spray coating, as reported in the manuscript. Before using the ink, it was sonicated for 10 min.

### PEDOT:PSS Ink Preparation

PEDOT:PSS ink was obtained starting from PEDOT:PSS Clevios PH 1000 from Heraeus. The bought PEDOT:PSS watery solution was initially diluted down to 50 mg/mL by using ethanol as a cosolvent in a water/ethanol solution. In particular, 50 mL of the starting watery solution of PEDOT:PSS (1 mg/mL) was mixed with 950 mL of ethanol, obtaining a final concentration of 0.50 mg/mL. The obtained ink was used to fabricate the PEDOT:PSS coating reported in the manuscript. Before using the ink, it was sonicated for 10 min.

### Spray Coating Processes

PE substrates were first washed in ethanol for 30 s and then dried under nitrogen flux. Then, the PE substrates were cut into 1.5 cm x 1.5 cm pieces and UV-ozone treated for 10 min.

The spray coating was performed by using a homemade setup consisting of a cheap commercial airbrush, a sample holder rotating at about 30 rpm (a simple rotor engine) to increase the coating uniformity, a hand-held airbrush fixed on a stand, and a supplementary air flux source to increase solvent evaporation after spraying. The airbrush is obliquely oriented with a 45° angle with respect to the table, and the rotating sample holder is perpendicular to the airbrush. The airbrush trigger is fixed, and the compressed air is set at 1 bar, removing any reliability issues that may be caused by a manual control of the system. The distance between the airbrush and the sample plane can be changed according to the solvent or the desired spray area.

In the case of the graphene antifog treatment, the samples were spray-coated at different deposition times by using a customized setup (as sketched in Figure 1). The spray coating was then obtained by using the previously described spray coating setup and a distance of 13 cm between the airbrush and the rotating sample holder was selected to ensure that the spray cone effectively covered the entire sample holder, guaranteeing multiple samples deposition, avoiding the formation of ink drops on the samples. Following the film deposition, the samples were photonic annealed by using a xenon pulse light source (530–1400 nm) with <1.5 ms flash duration and a dose of 2.5–5 J cm^–2^ for three repetitions, following an optimization of this process in previous reports.^52^ The photonic annealing is designed to achieve carbonization of PVP increasing the electrical conduction, avoiding thermal annealing where a temperature >180 °C, required to remove the PVP, would damage the PE (melting point 90–110 °C). However, some samples without photonic annealing were prepared as a reference. The samples were then allowed to dry inside a chemical fume hood, and this coating is named graphene-PVP or graphene coating along the manuscript and represents the proposed graphene-based antifog treatment. The PE films that were graphene-coated and photonically annealed are briefly named G-PE.

In the case of graphene–PVP/PEDOT:PSS multilayer coating, the samples were initially spray-coated for 1 min and 30 s by using the same setup previously described. A distance of 20 cm (increased compared to the graphene spray) between the airbrush and the substrate was selected. The PEDOT:PSS ink is not completely made of ethanol, and 5% of water is still present. Thus, the spray’s distance is increased to avoid the formation of ink drops on the samples. The samples were then let dry inside the chemical fume hood, and this coating is named PEDOT:PSS coating along the manuscript. After the PEDOT:PSS coating, a spray coating of graphene was carried out for 30 s, realizing the final multilayer structure that is named the graphene/PEDOT:PSS coating in the paper and represents the proposed smart antifog treatment based on graphene. The PE film with this graphene/PEDOT:PSS coating is named as G/P-PE.

### Characterization Methods

The graphene ink, bare PE, PE with commercial antifog treatment, and all the realized G-PE, PEDOT:PSS-coated PE, and G/P-PE samples were characterized by UV–vis transmission and absorption spectroscopy using an Agilent Cary 60 UV–vis. We used the Beer–Lambert law, *A* = α*cl*, to obtain the flake concentration (*c*) in the graphene ink from the absorbance (measured at 660 nm by the UV–vis). In this equation, *l* represents the light path length that depends on the quartz cuvette dimension (10 mm in our case), while the value of the absorption coefficient at 660 nm, α = 2240L/g, was taken from the literature.^44^ UV–vis spectra were acquired on three different points across one sample, and the average data with the corresponding errors are shown.

Raman measurements were performed by a micro-Raman spectrometer (Renishaw 1000) by using a laser excitation at 514.5 nm, a ×100 objective (NA = 0.9), and an incident power of ≈3 mW. Raman fit of G and 2D graphene peaks was obtained by a Lorentz fit^62^ and was carried out by using the software OriginLab. Raman measurements were carried out on one sample.

Contact angle measurements were realized by First Ten Angstroms (FTA1000B) by using water droplets at ambient temperature in volumes of 5 μL on the bare PE, commercial antifog-treated PE, and graphene–PVP-coated PE. Contact angle measurements were carried out on three different points of one sample. The average contact angle and its standard deviation are reported in the manuscript. For the contact angle measurement as a function of time, the measure is repeated on the same sample, reporting the error on three measurements for the period.

Tensile elongation tests were performed by using a Linkam MFS—modular force stage. All the samples were prepared in a bow-tie shape according to the instrument’s instruction, with a sample length of 1.5 cm between the two moving clamps. More than three samples of each type were characterized, acquiring the stress as a function of the strain that allowed measurement of the Young’s modulus (elastic response of the material), yield strength (relative to the elastic-to-plastic transition), and ultimate strength (maximum stress supported by the material). Tensile test measurements were carried out on one sample of each type.

The same spray coating setup described in the spray coating process section was used to test the graphene antifog treatment on PE under continuous water exposure for 1 h. The samples were put under rotation at 30 rpm, sprayed with a compressor pressure of 2 bar, at a distance of 10 cm, and with 2 bar of air pressure pointed onto their surface. In such a way, we ensure a large formation of water drops moving onto the sample that is used to attest to whether the coating material is stable or not in water. During this test, the transmittance of the samples was acquired to attest to whether it has changed. For an unstable coating, redispersions of graphene flakes in water should occur. Hence, a negligible transmittance change means nonredispersions and that the coating is stable under water exposure. G-PE films with and without photonic annealing were tested at the same time to guarantee the consistency of the results. Exposure time to the water mist was investigated in the range between 5 min and 1 h. The integrated *T* (400–700 nm) normalized to one of the uncoated PE was acquired and compared. The UV–vis measurement is repeated on three points of the same sample for each time of water treatment, reporting the average integrated *T* and the obtained error.

Current–voltage (I–V) and sheet resistance measurements of all of the samples were carried out by using a Karl Suss/Microtec PMF8 manual probe station coupled with a Keithley 4200-SCS semiconductor characterization system. This measurement was performed on one sample. In particular, the sheet resistance was measured at room temperature in a Van der Pauw configuration for three different times, reporting the average value and the associated error. Bending tests were carried out by varying the angle between different positions while simultaneously measuring the resistance via copper pads at the edge of the sample. This test has been performed on one sample, repeating the bending cycle 10 times.

Thermoelectric measurements were carried out by using a Keithley 2200-30-5 as the power source for the G/P-PE samples in the bias range of 0–30 V, acquiring at the same time the IR images with the temperature map by using a FLIR ThermoCam B4 as an infrared camera. The measurements were performed on one sample, reporting the average temperature and error on three points measurement.

Steady-state thermal conductivity measurements (SSM) were conducted using a physical property measurement system (PPMS, DynaCool System, Quantum Design), equipped with the thermal transport option (TTO, DynaCool CAN-based D670, Quantum Design).^69^ Samples were mounted on a TTO puck in a four-probe lead configuration, with leads connected to a resistive heater, two temperature sensors, and a heat sink (Figure 6a). Conductive silver paint (SCP03B, Electrolube) was applied at the lead-sample interfaces to minimize thermal contact resistance. The puck was then enclosed in a copper isothermal shield to mitigate radiative heat losses, and all measurements were performed under ultrahigh vacuum conditions to ensure minimal thermal interference. Thermal conductivity (κ) was determined at three temperatures, 278, 303, and 328 K, by using the steady-state heat conduction equation: κ = −qΔx/ΔT, where q represents the steady-state heat flux input, and Δ*T* is the temperature gradient across a specified sample length (Δ*x*), recorded once thermal equilibrium was reached. These measurements were performed on three different points. Corrections for radiative and parasitic heat losses were applied, accounting for system influences and sample-specific parameters, such as emissivity, cross-sectional area, and surface area. Detailed experimental procedures are provided in the Supporting Information.

## Supplementary Material



## Notes

Adhesion test of graphene coating, description of the steady-state method (SSM) and analysis of thermal conductivity measurement , and description of the emissivity measurements of thin films (PDF)

## #

A. A. L. is currently at the Department of Chemical, Biological, Pharmaceutical, and Environmental Science, University of Messina

## ABBREVIATIONS

LPE: liquid-phase exfoliation
PE: polyethylene
PVP: polyvinylpyrrolidone
**PEDOT:**PSS: poly­(3,4-ethylenedioxythiophene)-poly­(styrenesulfonate)
G-PE: graphene coating on a PE film
G/P-PE: graphene/PEDOT:PSS multilayer coating on a PE film
SSM: steady-state method
PPMS: property measurement system
TTO: thermal transport option
