# Supplementary material for: Sprayed Graphene-Based Coating Enabling Antifog and Smart Features
Source: ACS Appl Mater Interfaces. 2025 Jun 13;17(25):37012–24. doi: 10.1021/acsami.5c05680 (PMC12203466; doi:10.1021/acsami.5c05680)
Supplement: Supplementary file 1 [file am5c05680_si_001.pdf]

Supporting Information

Sprayed graphene-based coating enabling antifog and smart features

Antonio Alessio Leonardi[1,2,†], Teresa Berges Acosta[3], Hongyang Dang[1,2], Sihui Liu[3], Chengning Yao[3], and Felice Torrisi[1,2,3,]*

1 Dipartimento di Fisica e Astronomia, Università di Catania, Via Santa Sofia 64, 95123 Catania, Italy;

2 CNR-IMM, Catania University Unit, Via Santa Sofia 64, 95123 Catania, Italy

3 Department of Chemistry, Molecular Sciences Research Hub, Imperial College London, London, W12 0BZ UK & Centre for Processable Electronics, Imperial College London, London, SW7 2AZ UK

*corresponding author felice.torrisi@unict.it, f.torrisi@ic.ac.uk;

†Antonio A. Leonardi is currently at the Department of Chemical, Biological, Pharmaceutical, Environmental Science, University of Messina.

Adhesion test of graphene coating

The adhesion of the graphene coating on the PE sheet was tested following the ISO 2409 procedure. ISO 2409 describes a cross-cut test designed to assess the adhesion of thin films or coatings to a substrate. In this method, a cutting tool is used to score a grid pattern on the coating, after which an adhesive tape is applied and then peeled off from the grid area. The adhesive tape and the grid are then visually inspected, and adhesion is classified on a scale from 0 to 5, based on the presence and extent of flaking along the intersections, edges, and cuts.

As visible by the Fig. S1 reporting the adhesion test an excellent classification of 1 can be attributed to the fabricated coating as no coating detachment is attested on the tape and only slight flaking at the intersections is attested on the grid.

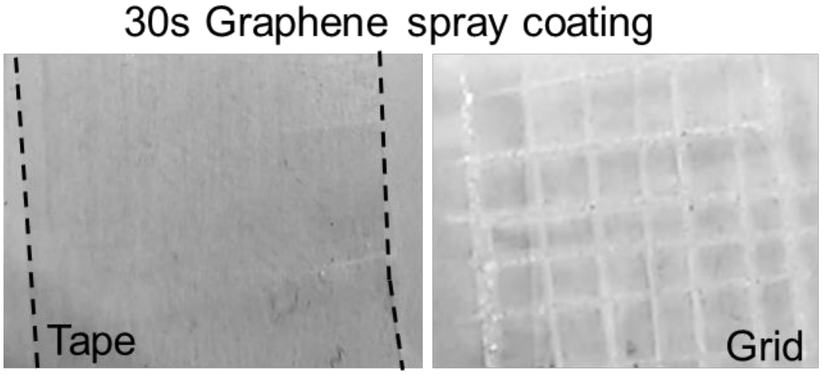

Figure S1. Adhesion test results for the 30s graphene spray coating

Description of the Steady-State Method (SSM) and Analysis of Thermal Conductivity Measurement

The thermal conductivity of polyethylene (PE) films, PEDOT:PSS-coated PE (PEDOT-PE), graphene-coated PE (G-PE), and multilayer graphene/PEDOT:PSS-coated PE (G/P-PE) films was determined using the steady-state (SSM) method. Measurements were conducted using a Quantum Design Physical Property Measurement System (PPMS, DynaCool System, Quantum Design)

equipped with the Thermal Transport Option (TTO, DynaCool CAN-based D670, Quantum Design) [1].

Sample preparation and mounting

The samples were prepared by rolling each film into a cylindrical configuration and mounting them on a TTO puck in a four-probe configuration. As illustrated in Figure S2, four copper leads were attached to the sample, establishing connections to a resistive heater (heat source) at the top, two temperature sensors (hot and cold thermometers) to measure the temperature gradient, and a cold foot (thermal sink) at the bottom. Conductive silver paint (SCP03B, Electrolube) was then applied at the lead-sample interfaces to minimize thermal contact resistance. Once mounted, the sample assembly was enclosed in a copper isothermal shield to reduce radiative heat losses. Measurements were conducted at three temperatures - 278 K, 303 K, and 328 K – under ultrahigh vacuum conditions.

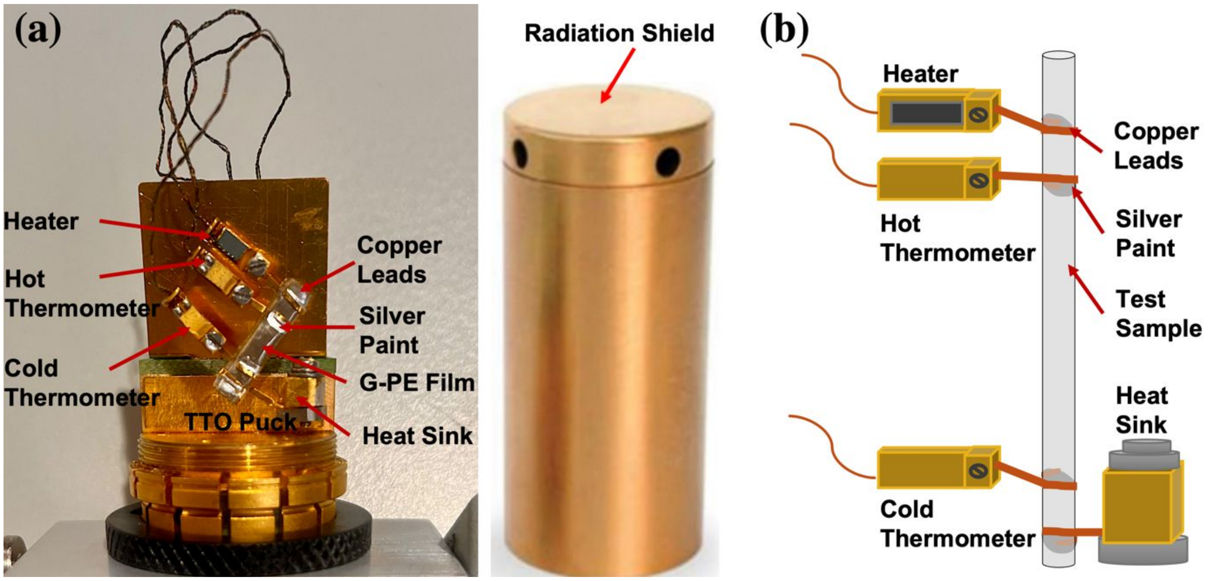

Figure S2. (a) TTO puck with a mounted sample and its copper radiation shield. (b) Schematic of the four-probe configuration, illustrating the connections between the sample and the leads [1].

Measurement Principles

The TTO module employs the steady-state heat flow method to determine the thermal conductivity (κ) of the samples. A known heat input (q) is applied to the top of the sample via the resistive heater, while the bottom is maintained at a constant temperature through the thermal sink. The temperature gradient (ΔT) across a specified sample length (Δx) is recorded by the thermometers once the system reaches thermal equilibrium. The thermal conductivity is then calculated using the following relation [2]:

$$\kappa = (\Delta x/A)q/\Delta T \quad (1)$$

Where, κ is the thermal conductivity (W/m·K), q is the power input from the heater (W), A is the cross-sectional area of the sample (m^2), Δx is the distance between the thermometers (m), and ΔT is the temperature gradient across a specified sample length (K).

To account for radiative and parasitic heat losses, the system applies additional corrections using the following equation (2) [1]:

$$\kappa_{sample}\Delta x_{sample}/\Delta T_{sample} = P_{cond} = P_{heater} - P_{rad} - P_{loss} \quad (2)$$

Where, P_{cond} is the conductive heat flow through the sample, P_{heater} is the total power supplied by the heater, P_{rad} is the radiative heat loss, and P_{loss} accounts for parasitic heat losses through wires, sensors, and other components.

Radiative heat losses (P_{rad}) are estimated using the Stefan-Boltzmann law as follows (3):

$$P_{rad} = 2\sigma_T S\varepsilon \cdot T^3 \cdot (\Delta T_H + \Delta T_C) \quad (3)$$

where σ_T is the Stefan-Boltzmann constant ($\sigma_T = 5.67 \times 10^{-8} W/m^2K^4$), S is the sample surface area (m²), ε is the sample emissivity (determined experimentally, see SI2), T is the average puck temperature (K), and T_H and T_C are the average temperatures of the hot and cold thermometers during measurements (K).

Parasitic heat losses (P_{loss}) are estimated using an empirically determined polynomial equation (4):

$$P_{loss} = d(aT + bT^2 + cT^3)\Delta T_{loss} \tag{4}$$

where, a, b, c, and d are empirically determined constants, and ΔT_{loss} is the average temperature change in the wires, heaters, and sensors, calculated by the hot thermometer readings once thermal equilibrium is reached (change within 0.1 %).

Input Parameters

Table 1 summarizes the key geometry parameters and emissivity values used in the thermal conductivity calculations for each sample.

Table S1. Geometry parameters and emissivity values for each sample.

Sample	Δx (mm)	S (mm2)	A (mm2)	$\boldsymbol{\varepsilon}$
PE	7	71.4	2	0.93
PEDOT-PE	9	90	2	0.88
G-PE	6	48	2	0.80
G/P-PE	8	56	2	0.85

Description of the Emissivity Measurements of Thin Films

The emissivity of the thin-film samples, including polyethylene (PE) films, PEDOT:PSS-coated PE (PEDOT-PE), graphene-coated PE (G-PE), and multilayer graphene/PEDOT:PSS-coated PE (G/P-PE), was measured using a Contact Thermometer Method (CTM) in accordance with the ASTM E1933 Standard. This method was adapted from a previously reported procedure [3].

Experimental Setup

The custom-built setup for emissivity measurements is shown in Figure S3(a). The setup consists of a high-precision hot plate (Stuart SD160), an infrared (IR) camera (UNI-T UTi120S, Spectral range of λ = 7.5–14 μm) positioned perpendicularly (90˚) above the sample at a distance of 40 cm, K-type thermocouples connected to a data logger (TASI TA612C) for precise temperature monitoring, and a cardboard shield to minimize interference from ambient light.

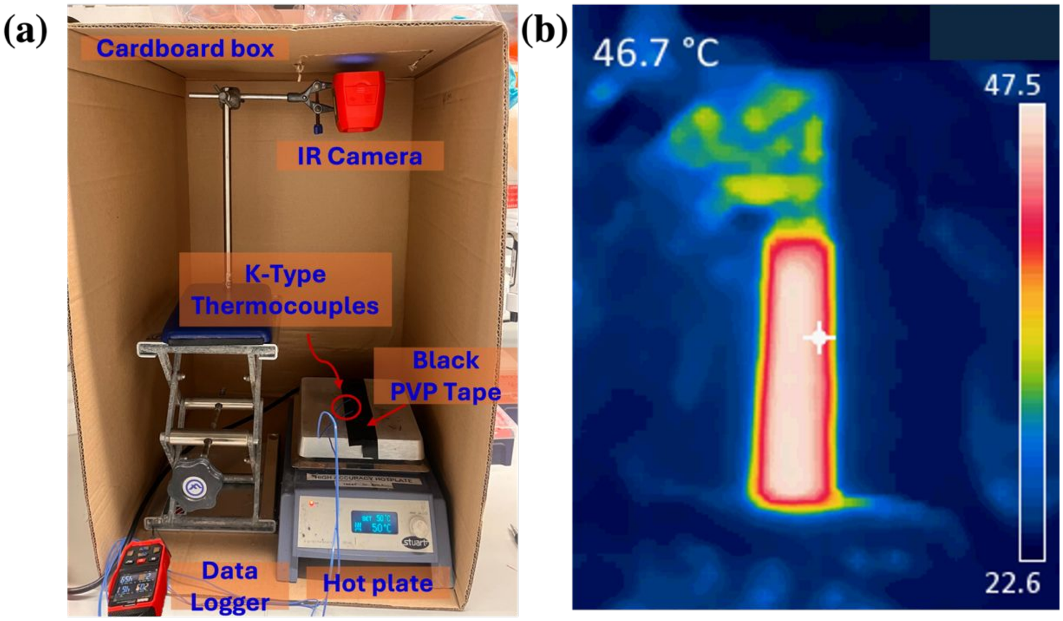

Figure S3. (a) Custom-built setup used for emissivity measurements. (b) IR camera image of the reference black PVC tape at an emissivity setting of 0.95, showing strong consistency between the temperature recorded by the camera and the thermocouple (≈46.3 ± 1 ˚C).

Measurement Procedure, Validation, and Calibration

Emissivity measurements were conducted by placing the samples on a hot plate set to a constant temperature of 50 °C. K-type thermocouples were placed in direct contact with the film surfaces at multiple locations to measure the actual sample temperature after thermal equilibrium was reached. Simultaneously, an IR camera was used to record the surface temperature of the samples. The emissivity setting of the IR camera was manually adjusted until its temperature readings matched those of the thermocouples. The adjusted emissivity values were recorded as the estimated emissivity of the samples within the spectral range of the IR camera.

To validate the accuracy of the measurement procedure, black PVC tape —known to have an emissivity of 0.95—was used as a reference material [3]. Figure S4(a–c) presents a comparison of temperatures measured by the IR camera at emissivity settings of 0.5, 0.8, and 0.95. When the IR camera's emissivity was set to 0.95, the temperature readings closely matched the thermocouple measurements ($\approx 46.3 \pm 1$℃), confirming the reliability of this procedure. As such, PVC tape was periodically employed to recalibrate the IR camera throughout the experiment.

Despite its reliability, the method has certain limitations. Direct contact between the thermocouples and the samples may cause localized cooling effects, potentially altering the measured surface temperature. Additionally, the hot plate may introduce spatial temperature nonuniformities (hot spots) and temporal fluctuations. To minimize these effects, measurements were repeated three times for each sample, following ASTM standard guidelines. Furthermore, emissivity measurements were performed on 3–5 films per sample type to ensure reproducibility. The final emissivity values were determined by averaging all measurements: $\varepsilon \approx 0.933 \pm 0.006$ for PE films, (consistent with previously reported values [4,5]); $\varepsilon \approx 0.880 \pm 0.014$ for PEDOT-PE films; $\varepsilon \approx 0.807 \pm 0.015$ for G-PE films; and $\varepsilon \approx 0.848 \pm 0.021$ for G/P-PE films (Figure S4(d-f)).

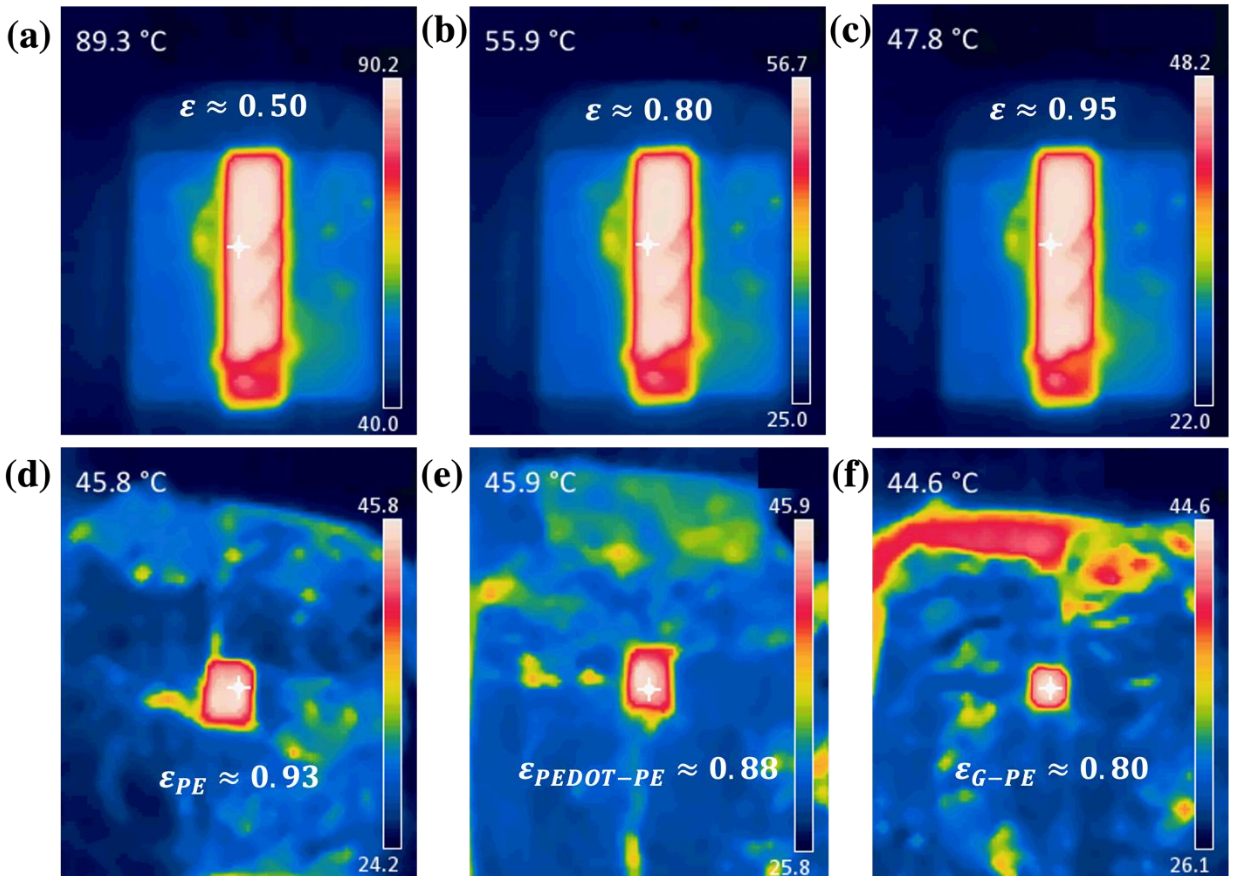

Figure S4. IR-camera images of PVC tape at emissivity settings of (a) 0.5, (b) 0.8, and (c) 0.95. IR-camera images of (d) a PE film at an emissivity setting of 0.93, aligning with the thermocouple's temperature (≈45.8 ± 1 ˚C); (e) a PEDOT-PE film at an emissivity setting of 0.88, consistent with the thermocouple's temperature (≈45.9 ± 1 ˚C); and (f) a G-PE film at an emissivity setting of 0.80, closely matching the thermocouple's temperature (≈44.5 ± 1 ˚C).

References

(1) Yao, C.; Leahu, G.; Holicky, M.; Liu, S.; Fenech-Salerno, B.; Lai, M. C.; Larciprete, M. C.; Ducati, C.; Divitini, G.; Voti, R. L.; Sibilia, C.; Torrisi, F. Thermally Conductive Hexagonal Boron Nitride/Polymer Composites for Efficient Heat Transport. *Adv Funct Mater* 2024, *34* (46), 2405235. https://doi.org/10.1002/ADFM.202405235.

(2) *Physical Property Measurement System Thermal Transport Option User's Manual*. https://web.njit.edu/~tyson/PPMS_Documents/PPMS_Manual/1684-100%20Rev.%20B0%20TTO.pdf (accessed 2024-12-22).

(3) Leone, C.; Genna, S.; Bertocchi, F.; Giordano, M.; Martone, A. A Procedure to Measure the Emissivity of Ultra-Thin Graphene Based Film in Long Wavelength Infrared (LWIR) Spectrum Region. *Opt Laser Technol* 2021, *138*, 106910. https://doi.org/10.1016/J.OPTLASTEC.2020.106910.

(4) *Infrared Plastic Temperature Measurements | IMPO*. https://www.impomag.com/maintenance/article/13216904/infrared-plastic-temperature-measurements (accessed 2024-12-22).

(5) *Surface Emissivity Coefficients*. https://www.engineeringtoolbox.com/emissivity-coefficients-d_447.html?utm_source=chatgpt.com; (accessed 2024-12-22).
